# 814. Impact of Testing Turnaround Times on Clinical Outcome in Hospitalized Patients with Histoplasmosis and Blastomycosis

**DOI:** 10.1093/ofid/ofad500.859

**Published:** 2023-11-27

**Authors:** Liam Dalton, Blair Richards, Marisa H Miceli

**Affiliations:** University of Michigan Medical School, Ann Arbor, Michigan; University of Michigan Medical School, Ann Arbor, Michigan; University of Michigan, Ann Arbor, Michigan

## Abstract

**Background:**

Patients (pt) with histoplasmosis (histo) and blastomycosis (blasto) requiring hospitalization have unacceptably high mortality rates. In part, this is due to a delay in diagnosis. Serum (S) and urine (U) antigen (Ag) testing are often crucial for timely antifungal therapy. Clinical observations suggested delays in ordering samples to be sent to reference labs upon admission and prolonged turnaround time (TAT) for test results can lead to delays in starting therapy and poor outcomes. We sought to determine the impact of TAT for histo and blasto Ag testing on clinical outcomes of hospitalized patients (pt) at our institution.

**Methods:**

Retrospective evaluation of all adult inpatients with histo or blasto diagnosed by positive U or S Ag tests from 1/1/15-12/31/22 was conducted. TAT from admission to result (TAT-AR) and from sample collection to result (TAT-CR) for the earliest positive test were calculated. Clinical data, including treatment & outcomes were collected. Only pt who received antifungal therapy or had a postmortem diagnosis were included. The relationship between TAT-AR and TAT-CR and outcome was evaluated.

**Results:**

Of 39 pt (mean age 47±19 yr), 26 (67%) were men and 33 (85%) were immunocompromised: solid organ transplant recipients, 15; hematologic malignancy, 3; autoimmune/inflammatory disease on treatment, 12; others, 3. Thirty-five pt had pulmonary (8) or disseminated histo (27); 4 had disseminated blasto. Nine pt (23%) died within 4 months of diagnosis, including 8 pt with histo and 1 with blasto. Mean TAT-AR for these 9 deceased pt was 14.7± 19.5 days (d) vs 6.1±3.1 d for 30 survivors (p=.02). Mean TAT-CR for these 9 deceased pt was 5.4±1.5 d vs 3.9±1.3 d for 30 survivors (p=.003). Of the 32 pt who required IV amphotericin B (29) or died before any antifungal therapy was given (3), mean TAT-CR was 5.4±1.5 d for those who died vs 3.7±1.1 d for those who survived (p=.0013). ICU transfers, 30-d readmissions, and length of stay were not impacted by TAT-AR or TAT-CR.

**Conclusion:**

Delays in TAT for initiating testing and obtaining results were associated with increased mortality of hospitalized pt with newly diagnosed histo or blasto. These findings highlight the importance of early testing and timely test results to guide treatment for hospitalized pt with these diseases.

**Disclosures:**

**Marisa H. Miceli, MD**, Astellas: Advisor/Consultant|F2G: Grant/Research Support|Scynexis: Advisor/Consultant|Scynexis: Grant/Research Support

